# Characterisation and Classification of Foodborne Bacteria Using Reflectance FTIR Microscopic Imaging

**DOI:** 10.3390/molecules26206318

**Published:** 2021-10-19

**Authors:** Jun-Li Xu, Ana Herrero-Langreo, Sakshi Lamba, Mariateresa Ferone, Amalia G. M. Scannell, Vicky Caponigro, Aoife A. Gowen

**Affiliations:** 1 School of Biosystems and Food Engineering, University College Dublin, Belfield, Dublin 4, Ireland; junli.xu@ucd.ie (J.-L.X.); ana.herrero-langreo@ucd.ie (A.H.-L.); vicky.caponigro@ucd.ie (V.C.); 2 School of Agriculture and Food Science, University College Dublin, Belfield, Dublin 4, Ireland; sakshi.lamba@ucdconnect.ie (S.L.); mariateresa.ferone@ucd.ie (M.F.); amalia.scannell@ucd.ie (A.G.M.S.); 3 Institute of Food and Health, University College Dublin, Belfield, Dublin 4, Ireland

**Keywords:** FTIR, foodborne bacteria, classification, machine learning, stainless steel

## Abstract

This work investigates the application of reflectance Fourier transform infrared (FTIR) microscopic imaging for rapid, and non-invasive detection and classification between *Bacillus subtilis* and *Escherichia coli* cell suspensions dried onto metallic substrates (stainless steel (STS) and aluminium (Al) slides) in the optical density (OD) concentration range of 0.001 to 10. Results showed that reflectance FTIR of samples with OD lower than 0.1 did not present an acceptable spectral signal to enable classification. Two modelling strategies were devised to evaluate model performance, transferability and consistency among concentration levels. Modelling strategy 1 involves training the model with half of the sample set, consisting of all concentrations, and applying it to the remaining half. Using this approach, for the STS substrate, the best model was achieved using support vector machine (SVM) classification, providing an accuracy of 96% and Matthews correlation coefficient (MCC) of 0.93 for the independent test set. For the Al substrate, the best SVM model produced an accuracy and MCC of 91% and 0.82, respectively. Furthermore, the aforementioned best model built from one substrate was transferred to predict the bacterial samples deposited on the other substrate. Results revealed an acceptable predictive ability when transferring the STS model to samples on Al (accuracy = 82%). However, the Al model could not be adapted to bacterial samples deposited on STS (accuracy = 57%). For modelling strategy 2, models were developed using one concentration level and tested on the other concentrations for each substrate. Results proved that models built from samples with moderate (1 OD) concentration can be adapted to other concentrations with good model generalization. Prediction maps revealed the heterogeneous distribution of biomolecules due to the coffee ring effect. This work demonstrated the feasibility of applying FTIR to characterise spectroscopic fingerprints of dry bacterial cells on substrates of relevance for food processing.

## 1. Introduction

Foodborne bacteria are of major concern and pose a serious threat to public health and food safety. According to the World Health Organization, an estimated 2.2 million people die of foodborne diseases every year [1]. The rising awareness of the health risks of foodborne illnesses has accounted for the greater efforts to develop rapid and sensitive approaches for pathogen detection and identification [2]. Conventional culture-based methods have been a standard practice to detect foodborne bacteria/microorganisms for nearly one century. Such methods usually consist of microbiological culturing and isolation of the bacteria/microorganisms which is subsequently confirmed by biochemical and/or serological tests [3]. Despite being reliable, culture-based methods are labour-intensive as well as time-consuming, requiring 5–7 days to obtain the results. In this sense, results are most likely unavailable until the food product has been either released to the market or even consumed, which unavoidably accounts for the growing risk of transmission of pathogens [4]. Advances in new technologies have shown great promise in the rapid detection of pathogens; for instance, polymerase chain reaction (PCR) has become an important tool to identify pathogenic organisms in a variety of foods [5]. While PCR methods are sensitive in recognition of bacteria/microorganisms, these methods are usually expensive for routine use in common testing laboratories requiring substantial laboratory equipment and highly skilled personnel.

In a modern food-processing scenario, real-time monitoring procedures are desired to make timely corrective action. Vibrational spectroscopy coupled with machine learning algorithms has the potential to meet the requirements of being rapid, sensitive, and non-destructive [6]. Fourier transforms infrared spectroscopy (FTIR) probes structural information of biological molecules including carbohydrates, proteins, and lipids; therefore it has been proven to be useful in the analysis of bacteria [7,8]. For instance, FTIR using an attenuated total reflectance (ATR) setting has been applied to bacterial suspension in distilled water to study compositional and structural changes of bacteria during culturing [9], demonstrating the potential of FTIR spectroscopy in providing molecular fingerprints of the cell envelope, as well as compositional and metabolic information of the cytoplasm under different physiological conditions. Bağcıoğlu, Fricker [10] applied FTIR spectroscopy in combination with the artificial neural network for discrimination of *Bacillus cereus* group members, which accounted for 100% correct identification for the training set and 99.5% correct identification overall. In addition, vibrational spectroscopies in combination with microscopy enable the acquisition of high-quality micro-spectra from low sample amounts. In many cases, the reduced requirements for microbial biomass allow the characterization of bacterial cells without the need for microbial cultivation [11].

The main objective of this study was to investigate FTIR microscopic reflectance imaging for pixel-level classification between *Bacillus subtilis* (Gram-positive) and *Escherichia coli* (Gram-negative) with the aid of machine learning algorithms. *Bacillus subtilis* was a model organism to study endospores, i.e., tough protective structures that tolerate extreme preservation conditions, whilst *Escherichia coli* was considered as an indicator bacterium in food safety and hygiene. This work was a part of a larger project evaluating the capability of spectral imaging at multiple spatial scales (from microscopic to macroscopic) for the characterisation and classification of foodborne bacteria. Herein, we systematically investigated the performance of the FTIR reflectance imaging in terms of transferability and robustness. The limit of detection of the proposed FTIR approach was also estimated. We additionally investigated the suitability of two substrates (i.e., stainless steel and mirror aluminium slides) on collecting reflectance FTIR reflectance spectra.

## 2. Material and Methods

### 2.1. Sample Preparation

Type strains of *Bacillus subtilis* DSM 10 (*B. subtilis*) and *Escherichia coli* DSM 11,250 (*E. coli*) were acquired from the German Collections of Microorganisms and Cell Cultures (Braunschweig, Germany). Bacterial strains were recovered from −80 °C glycerol stock, suspended in 4 mL of tryptic soya broth/TSB (Oxoid, CM0129), and incubated overnight at 30 °C. Overnight cultures were resuspended in fresh TSB and grown to mid-exponential phase. The bacterial cultures were then harvested by centrifugation (5000 rpm for 15 min at 4 °C), washed twice each in sterile phosphate buffer saline/PBS (Gibco, Life Tech. 18912–014) as well as sterile water. These were then resuspended in sterile water to desired concentration levels at OD_600nm_ of 10, 1, 0.1, 0.01, and 0.001 using Shimadzu UV mini Spectrophotometer Model 1240. Finally, 10 µL of bacterial suspensions were deposited on the substrate in duplicates, dried for 20–30 min in a safety cabinet at room temperature, and stored at 4 °C prior to FTIR image collection. To estimate the number of viable cells in each sample, the plate count method was utilised wherein the samples were serially diluted to 10^−8^ in sterile water, cultivated 100 µL of each dilution in duplicates on Tryptic Soya Agar (TSA) followed by incubation at 37 °C for 24h. The numbers of colony forming units (CFU) per plate were recorded and converted to CFU/mL for the samples. As a conventional practice, colony counts in the range of 30–300 CFU were considered and when the reported mean count was less than the lowest acceptable level, the actual value was used.

Stainless steel AISI 316 finished 2B (STS) and polished mirror aluminium (Al) slides were both used. Stainless steel (Amari Ireland Ltd., Dublin, Ireland) is widely used in food manufacturing due to its high resistance to acids, alkalis, and chlorides, such as salt. The sample set (Table 1) consisting of four different levels of bacterial cell concentrations at OD_600nm_ i.e., 10, 1, 0.1, and 0.001 was generated in a span of several months (from January to September of 2020). Each concentration comprised 8 replicates. To evaluate the performance of mirror Al slides as substrate (ALUM EZ-SPOT MICRO MOUNT, Thermo Fisher Scientific, Madison, USA), 2 biological replicates of each strain were prepared on 23 and 24 September 2020, covering the concentration of 10, 1, 0.1, 0.01, and 0.001 OD. Two drops of each replicate were deposited and scanned, leading to 4 images of each concentration.

### 2.2. FTIR Image Acquisition

Spectral images were collected in the reflectance mode using a Thermo Scientific^TM^ Nicolet^TM^ iN10 Infrared Microscope (10× magnification, mercury-cadmium-tellurium cooler detector). The system was purged with gas nitrogen overnight before use and then maintained under purge during scanning. The detector was cooled with liquid nitrogen and the infrared spectra were captured in the spectral range of 4000–675 cm^−1^ with a resolution of 4 cm^−1^, at an average of 4 scans per pixel. Both the aperture size and step size measured 200 × 200 µm for all scans. Samples were measured in reflectance and calibrated using a gold disc placed on the standard sample plate. FTIR microscopic images of BS and EC at concentrations ranging from 0.1 OD to 10 OD are shown in Figure S1 of the Supplementary Materials.

### 2.3. Spectral Pre-Processing

All spectral data analysis and modelling were carried out in MATLAB computing environment (release R2020a, The MathWorks, Inc., Natick, MA, USA) incorporating functions from Statistics and Machine Learning Toolbox, Image Processing Toolbox and additional functions written in-house. For a visual comparison of spectra, asymmetric least squares smoothing [12,13] was implemented to alleviate inconsistent baselines. A second-order polynomial was fitted to all data points of each spectrum, followed by the estimation of the baseline in an iterative manner. The next step was to subtract the estimated baseline from the original spectrum. In the present case, the smoothing and weighting (penalizing) parameters were determined as 10^6^ and 0.005, respectively.

For the data used to build classification models, smoothing followed by standard normal variate (SNV) was implemented. Smoothing was applied on the pixel spectrum using a Savitsky Golay filter (window size = 15 points and the polynomial order = 3). Afterwards, SNV was used to reduce multiplicative interferences.

### 2.4. Background Removal

For both substrates, background removal was carried out as the first step in data analysis. Principal component analysis (PCA) was performed on the SNV pre-processed spectra in the 4000–3600 cm^−1^ range, which highlighted the major differences between the substrate and bacterial pixels. Afterwards, segmentation was conducted by manual thresholding of the PC1 score image. The background elimination on aluminium slides was more complex. There were 12 wells per slide and bacterial samples were deposited in individual wells, as shown in the Supplementary Materials (Figure S2). The first procedure was to segment the region corresponding to the wells by thresholding on PC1 images, followed by another thresholding to separate bacterial pixels from the mirror aluminium slide within the well. Only pixels segmented as representing bacteria were extracted and used for the subsequent model development. Table 2 illustrates the number of pixels of each bacterial strain for each concentration. The number of pixels on Al is much lower than that on STS due to fewer replicates for the Al substrate, i.e., 2 replicates (4 drops) of each bacterial strain for Al and 8 replicates for STS.

### 2.5. Classification Modelling

Partial least squares discriminant analysis (PLSDA)[14] and support vector machine (SVM) were used to build models for the discrimination of bacterial strains. As a common practice, spectral images were “unfolded” prior to model development, which simply refers to rearranging data with three dimensions [(1) rows, (2) columns, and (3) wavelengths] to a matrix with two dimensions [(1) rows × columns against (2) wavelengths]. After eliminating background, pixels were extracted from different spectral images and concatenated to make a matrix (**X**) where the rows standing for observations and columns for spectral features. A categorical label was assigned for each class: ‘1’ was assigned for *B. subtilis* and ‘2’ for *E. coli*. A dummy matrix (**Y**) was created where the rows represent observations and columns represent the true classes. Model output was a predicted value (either ‘1’ or ‘2’) for an unknown pixel. PLSDA and SVM models were developed from **X** and **Y**. Venetian blinds cross-validation was applied to determine the optimal number of latent variables (LVs) [15], by checking the evolution of the accuracy with the number of LVs. The SVM classifier was trained using the kernel function of linear and sequential minimal optimization.

In this work, two modelling strategies were designed to evaluate model performance, transferability, and consistency among concentration levels. Modelling strategy 1 was proposed to develop a practical classification model that could classify bacterial samples at various concentrations. To achieve this, the model was built from a training set comprising half of the sample set of all concentrations ranging from 0.1 OD to 10 OD (concentrations lower than 0.1 OD were excluded because of inferior spectral quality, as discussed later in Section 3.1 and Section 3.2). For samples deposited on STS (see Table 1), the first 4 replicates of each concentration were grouped forming the training set, while the remaining served as the test set. In detail, this training set consists of 3218 pixels (1672 pixels of *B. subtilis* and 1546 pixels of *E. coli*) and the test set is comprised of 2864 bacterial pixels (1426 pixels of *B. subtilis* and 1428 of *E. coli*). With respect to samples deposited on Al, the first 2 replicates of each concentration were used as the training set, leading to 1013 bacterial pixels, while the test set consisting of the rest 2 replicates has 1192 pixels. Afterwards, the aforementioned best model developed from one substrate was tested on the bacterial samples from the other substrate, in order to evaluate the model transferability.

Modelling strategy 2 was devised to investigate if the spectral difference between two bacterial strains is consistent at various concentration levels. If true, the model built from one concentration should adapt to the bacterial samples collected at other concentrations. To be specific, for each substrate, models were developed using only one concentration level and tested on the other concentrations.

The performance of each binary classifier is assessed by the overall accuracy (OA) and Matthews correlation coefficient (MCC), a reliable statistical rate that demonstrates a high score only if the prediction obtained good results in all aspects. In addition to this, the sensitivity and specificity of classifying *B. subtilis* are also included. Sensitivity (also known as the true positive rate) assesses the proportion of positives that are correctly identified as such, while specificity (also named as the true negative rate) measures the proportion of negatives that are correctly identified as such. In addition, classification maps are generated to visualize the distribution of the correctly classified and misclassified pixels.

## 3. Results and Discussion

### 3.1. FTIR Spectra on Stainless Steel

**Figure 1:**
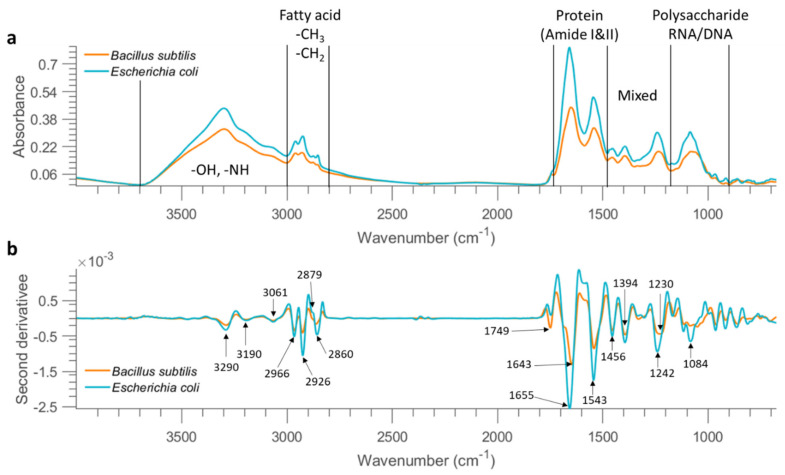
Mean FTIR spectra (after applying asymmetric least squares smoothing to remove baseline) of all bacterial cells from the concentration of 10 OD deposited on stainless steel (**a**) and the result after performing second derivative (**b**). The characteristic peaks are pointed out in the derivative spectra.

Mean FTIR spectra of all dry bacterial cells collected from the stainless steel substrate at the concentration of 10 OD were obtained and the baseline was removed by performing asymmetric least squares smoothing, with the resultant spectra presented in Figure 1a. As can be seen, FTIR spectroscopy provides mid-infrared spectral fingerprints of bacterial cells, originating from the different functional groups related to proteins, lipids, and carbohydrates. The acquired FTIR spectra of microbial cells are a superposition of contributions from all biomolecules found in a cell, making it challenging to find out the specific contribution from any particular biomolecules or molecular groups. The entire spectral range can be subdivided into five non-overlapping sub-regions according to specific chemical constituents, as shown in Figure 1a.

A broad band with a peak at 3300 cm^−1^ corresponds to O–H and N–H stretching vibrations. The 3000–2800 cm^−1^ region is dominated by the C–H stretching vibrations in fatty acids, as seen from Table 3 which lists band assignment based on the literature. The 1700–1500 cm^−1^ region represents amide I and amide II arising from various proteins and peptides. The 1500–1200 cm^−1^ region has multiple mixed contributions from proteins, fatty acids, and phosphate-carrying compounds, while the 1200–900 cm^−1^ region represents RNA/DNA with vibrations coming from PO^2−^ in combination with C–O–C stretching of polysaccharides in the cell wall [16].

The displayed spectra show similar patterns over the entire spectral range for *E. coli* and *B. subtilis*, suggesting similarity of the functional group chemistry of both types of bacterial cells studied. Overall, the mean spectrum of *E. coli* demonstrates stronger absorption across the whole wavenumber range. This observation is well supported by higher cell counts observed in selected samples of *E.coli* (5.75 logCFU/mL) as compared to *B.subtilis* (3.72 logCFU/mL) at 0.1 OD. Similarly, for samples prepared on 23 and 24 September 2020, the cell counts at 1 OD ranged between 7.91–7.85 logCFU/mL for *E.coli* while a lower viable cell count of 6.48–6.66 logCFU/mL was found for *B.subtilis* (Table S1 of Supplementary Materials). The differences between the nature of the two bacteria, their shape & size as well as their multiplication/growth cycles may have further contributed to these variations.

Savitzky–Golay second derivative transformation (window size = 25 points and the polynomial order = 3) was applied to the raw mean spectra at the concentration of 10 OD (as shown in Figure 1b) to enhance the separation of overlapping bands. The broad band in the 3500–3000 cm^−1^ range consists of three minor bands at 3290 cm^−1^, 3190 cm^−1^, and 3061 cm^−1^. A series of bands are also observed between 3000 and 2800 cm^−1^; bands at 2926 and 2860 cm^−1^ can be assigned to CH_2_ stretching, while 2966 and 2879 cm^−1^ can be related to CH_3_ stretching, according to the literature (Table 3). The intensity ratio of CH_3_ groups to CH_2_ groups is observed higher in *B. subtilis* based on the mean second derivative spectra (Figure 1b), possibly because Gram-negative bacteria differ physically from Gram-positive bacteria, the former having an additional (outer) membrane, leading to distinct differences in fatty acid chains [19]. As a further step, we obtained the second derivative spectrum for each pixel and computed the ratio of 2966 cm^−1^ (CH_3_ group) to 2926 cm^−1^ (CH_2_ group). A two-sample *t*-test confirmed that this ratio was significantly higher in *B. subtilis* than that of *E.coli* at 10 OD and 1 OD (*p* < 0.01). However, the ratio between the two bacterial strains was not significantly different when the bacterial concentration reached 0.1 OD (*p* > 0.01). The carboxylic groups of bacterial cells exhibit distinct bands at ~1749 cm^−1^ due to the C=O stretching. Observable differences between these two bacterial strains are found in amide groups showing two intense bands in the 1700–1500 cm^−1^ range, indicating variations in the composition and structure of proteins and peptides. Another distinct feature arises from asymmetric P=O stretch with a peak at 1242 cm^−1^ for *E. coli* and 1230 cm^−1^ for *B. subtilis.* In addition, this band, representing the contribution of phospholipids from the cell membrane, appears broader for *B. subtilis.* In addition, differences in terms of band shape and peak position are observable in the spectral region of 1200–1000 cm^−1^ due to the combined contributions from polysaccharides and nucleic acids.

Mean spectra of each replicate of 10 OD samples are also plotted in Figure S3 to examine the repeatability. These 8 replicates were obtained from 4 independent experiments (see details in Table 1). From Figure S3, it is evident that substantial spectral variation among replicates is observed, although spectral profiles are similar across the entire range. An observable peak shift appears in the amide I and II groups, suggesting molecular structure changes in proteins and peptides and thus significant variations in bacterial cells cultured from different experiments, which will pose a challenge for the subsequent classification.

One sample image was randomly selected from each concentration and the pixel spectra of this sample (without baseline correction) were extracted, as displayed in Figure S4. The pixel spectra of stainless steel are plotted in Figure S5 for comparison. Stainless steel presents no prominent spectral features except the baseline slope and environmental interferences. Infrared light scatters when interacting with the rough surface of stainless steel, and this scattering effect is greater at lower wavelengths, leading to a sloping baseline. The pixel spectra of bacterial cells are also influenced by this baseline effect. It is also found that spectra of bacterial cells especially from lower concentrations suffer from environmental interferences although efforts were made to maintain a stable environment (e.g., keep lab door closed during the whole scanning period, continuous gas nitrogen purging, background spectra collection and removal every 10 min). This is because atmospheric water vapour and carbon dioxide (CO_2_) strongly absorb in the infrared region and they can interfere in the spectral regions of 4000–3600 cm^−1^, 2500–2000 cm^−1,^ and 1650–1400 cm^−1^. Spectral profiles indicate that fewer pixels are found to show bacterial features as the concentration decreases. Particularly, pixels of 0.001 OD exhibit nothing but the spectra of stainless steel, suggesting that the FTIR instrumental detection limit has been reached at this concentration. In this sense, samples at 0.001 OD are excluded from the following analysis and modelling procedure.

Mean spectra of all dry bacterial cells for each concentration are plotted in Figure S6. Weaker absorption over the entire spectral region is evidenced as the concentration of bacterial cells drops. Due to the dominance of spectral features at 10 OD, the characteristic absorbance bands are unnoticeable for the mean spectra of lower concentrations. As a result, normalization is performed by dividing the intensity at 2926 cm^−1^ (CH_2_ stretching) and the outcome is shown in Figure S7. It is found that the mean spectrum of 0.1 OD is heavily affected by the environmental interference and the baseline correction is less satisfying. At 0.1 OD, amide groups within the spectral domain of 1700–1500 cm^−1^ are compromised by the occurrence of water vapour interference. Moreover, spectral features below 1200 cm^−1^ are less apparent at a lower concentration.

### 3.2. FTIR Spectra on Aluminium Substrate

**Figure 2:**
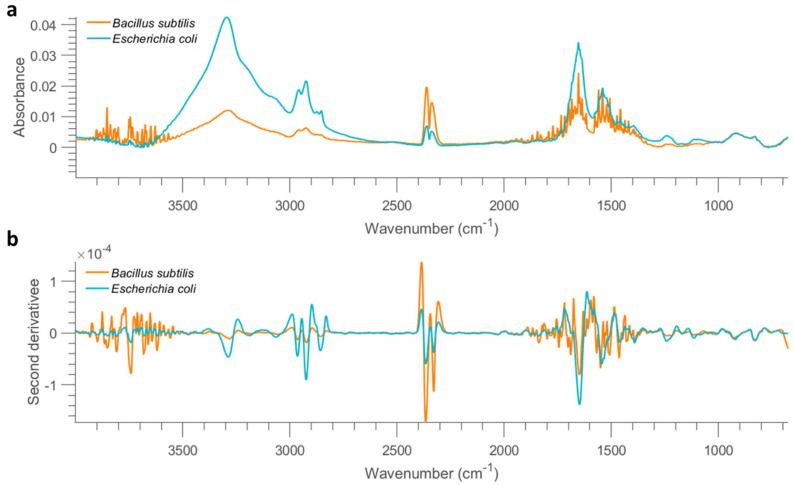
Mean FTIR (after applying asymmetric least squares smoothing to remove baseline) spectra of all bacterial cells from the concentration of 10 OD deposited on mirror aluminium slide (**a**) and the result after performing second derivative (**b**).

The mean spectrum of all dry cell samples acquired from the aluminium slide at 10 OD is shown in Figure 2a. Notably, weaker absorption is found for samples collected from the mirror aluminium slide, when compared with stainless steel. Specifically, the absorption of spectra collected from stainless steel is approximate 10 orders of magnitude higher than that of aluminium at the same bacterial concentration. This is because the smooth surface of mirror aluminium reflects much more infrared light than STS, leading to much lower absorption, which can be evidenced by much lower absorption of pixel spectra collected from empty mirror aluminium (Figure S8) compared to STS (Figure S5). It is also found that spectra of *B. subtilis* show strong atmospheric interferences, leading to poor spectral quality. Spectral profiles in the 3500–2500 cm^−1^ range, originating from O–H, N–H and C–H stretching are comparable to those obtained using the stainless steel substrate. Pixel spectra were extracted and displayed in Figure S9 to visualize the spectral profiles. As seen, serious atmospheric interferences are dominating the spectra when the concentration goes lower than 1 OD, again suggesting the deteriorate spectral quality compared to that from stainless steel. No pixels can be recognized as bacteria when the concentration is reduced to 0.01 OD. Therefore, similar to STS, samples with concentrations lower than 0.1 OD are not considered for further analysis.

### 3.3. Modelling Strategy 1

#### 3.3.1. Results from Stainless Steel Substrate

In order to identify the sensitive and effective spectral windows contributing to discrimination between *E. coli* and *B. subtilis*, classification models were separately developed in 4 regions: 4000–675 cm^−1^, representing the full spectral window measured; 1350–675 cm^−1^ and 3500–2600 cm^−1^, both of which are distinct from spectral regions sensitive to atmospheric changes; finally, since amide bands of the proteins in the cell are crucial for bacterial characterisation and identification [20], the 1722–910 cm^−1^ range was also included, as a compromise between spectral features from amide and the atmospheric interference. Prior to modelling, raw spectra were pre-treated (without baseline correction) by Savitzky–Golay smoothing (window size of 15 and the third-order polynomial degree) for alleviation of instrumental noise followed by SNV for reducing multiplicative effects.

**Figure 3:**
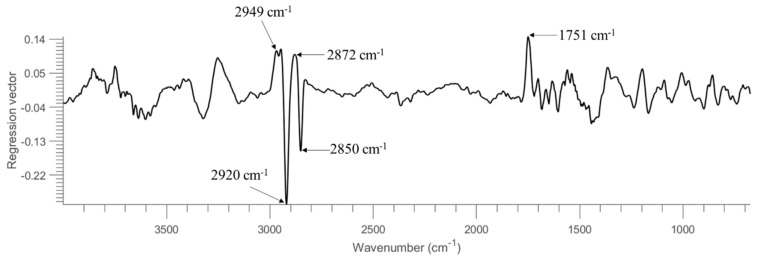
Regression vector of PLSDA model built from samples on STS using the entire spectral region.

To assess the generalization and robustness of the developed models, models were trained using half of the samples in the set and tested on the remaining half. That is, the model was built using pixel spectra obtained from the first 4 replicate images of each concentration (see Table 1). It should be noted that samples of 0.001 OD are not considered due to the absence of pixel spectra representing bacterial cells (see also Figure S4 & discussion in Section 3.1). To fairly compare machine learning strategies and different spectral regions, the overall accuracy (OA), MCC, sensitivity, and specificity were calculated from each model and summarized in Table 4. As seen, overall good performance can be witnessed in general, with accuracy around or higher than 90% in the test set. For PLSDA modelling, the use of the entire spectral region leads to an accuracy of 90% and MCC of 0.80, which is superior to using the spectral region of 1350–675 cm^−1^ or 1722–910 cm^−1^. Figure 3 displays the regression vector obtained from this PLSDA model. It can be noticed that the dominating spectral variables are found at 2949 cm^−1^, 2920 cm^−1^, 2872 cm^−1^, 2850 cm^−1^ and 1751 cm^−1^. The bands at 2949 cm^−1^ and 2872 cm^−1^, which can be respectively ascribed to ν(CH_3_) asymmetric and ν(CH_3_) symmetric vibrations of fatty acids (according to Table 3), have positive regression values. In contrast, the bands at 2949 cm^−1^ and 2872 cm^−1^, which can be respectively assigned to v(CH_2_) asymmetric and ν(CH_2_) symmetric vibrations of fatty acids, have negative regression values. The band of 1751 cm^−1^ relates to v(C=O) of lipid esters. It can be concluded that the important spectral variables contributing to the separation between *E. coli* and *B. subtilis* are associated with lipid compositions. The best spectral region for PLSDA modelling is then found using 3500–2600 cm^−1^, consistent with the regression vector (see Figure 3) where spectral variables in this spectral domain show high weightings. This model provides an accuracy of 94% and MCC of 0.89 for the test set. SVM outperforms PLSDA with an overall better modelling performance. Using the whole spectral region, 1350–675 cm^−1^ and 1722–910 cm^−1^ shows similar predictive ability, delivering an accuracy of 94% and MCC approximately around 0.88 for the test set. Once again, the SVM model developed from 3500–2600 cm^−1^ shows the best model performance with an accuracy of 96% and MCC of 0.93. In conclusion, the superiority of using the 3500–2600 cm^−1^ range can be evidenced, which is in accordance with Figure S8 where this spectral region shows good quality even at 0.1 OD concentration without obvious interference from the atmosphere.

**Figure 4:**
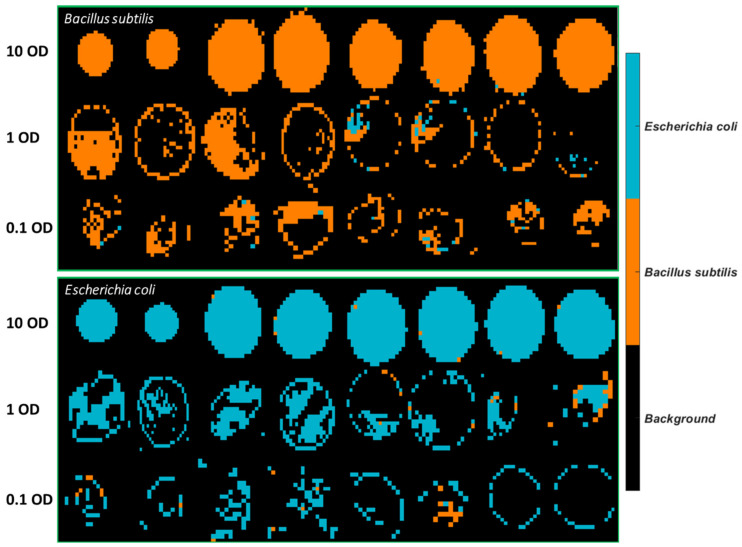
Classification maps obtained from the SVM model using 3500–2600 cm^−1^ for all samples deposited on STS. The training set consists of the first four images of each concentration, and the test set comprises the remaining four images of each set.

The best model obtained, i.e., the SVM model using spectral variables in the range of 3500–2600 cm^−1^, was applied to develop classification maps of each sample, as shown in Figure 4. Clearly, there are fewer misclassified pixels found on the 10 OD samples compared to lower concentrations. This is linked to the fact that the spectral signal of the bacterial cells is weaker at lower concentrations and more easily affected by atmosphere interference as also evidenced by the normalized mean spectral profiles in Figure S7.

#### 3.3.2. Results from Mirror Aluminium Substrate

For samples deposited on the aluminium substrate, since no pixel could be identified as a bacterial cell at 0.01 OD and 0.001 OD (see pixel spectra shown in Figure S9), these concentrations were not included in the modelling. The first two replicate images of each concentration belonging to the same biological replicate were used as the training set, leading to 1013 pixels, while the remaining samples were allocated to the test set with 1192 pixels. The results obtained using PLSDA and SVM classifiers and different spectral regions are summarised in Table 5. Overall, it is found that the developed model works well for the training set, yet the performance is much undesirable on the test set. This is probably because the training set consists of only one biological replicate, which makes it unable to capture the variability among different biological replicates. Again, the SVM modelling is superior to PLSDA with an overall better predictive ability. The regression vector of PLSDA using the entire spectral variables is not shown due to the inferior model performance (accuracy of 61% and MCC of 0.21 for the test set). The best model is found using 3500–2600 cm^−1^, both for PLSDA and SVM, which is consistent with the results from samples deposited on STS (see Table 4). In more detail, the best PLSDA model yields an accuracy of 76% and MCC of 0.53, while the best SVM produces an accuracy of 91% and MCC of 0.82.

**Figure 5:**
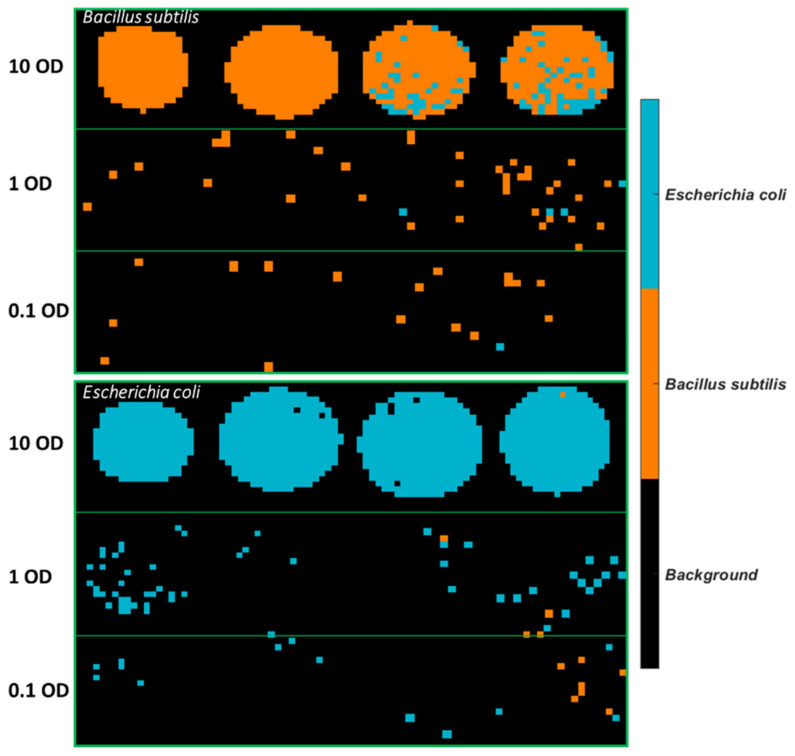
Classification maps obtained from the SVM model using 3500–2600 cm^−1^ for all samples deposited on Aluminium substrate. The training set consists of the first four images of each concentration, and the test set comprises the remaining four images of each set.

Classification maps of all images were generated using the SVM model built from 3500–2600 cm^−1^, as can be seen in Figure 5. It is observed that plenty of pixels of *B. subtilis* at 10 OD are incorrectly classified as *E. coli*, which explains the low sensitivity (0.82) in Table 5. As for *E. coli* samples, most misclassified pixels are found at 1 OD and 0.1 OD.

#### 3.3.3. Model Transfer between Substrates

This section aims to evaluate the model transferability between two substrates, i.e., stainless steel and aluminium slide. The best PLSDA and SVM models from Table 4; Table 5 are used for this purpose. Table 6 summarizes the modelling performance in terms of accuracy, MCC, sensitivity, and specificity. The PLSDA model built from samples on stainless steel presents moderate predictive ability with an accuracy of 71% and MCC of 0.43 when it is applied to the bacterial samples on the aluminium slide. Superior performance is found using SVM, producing an accuracy of 82% and MCC of 0.66 when transferring the STS model on the Al. It is also noticed that the sensitivity of predicting *B. subtilis* is much lower than the specificity (e.g., 0.61 versus 0.99)*,* suggesting the difficulty in identifying *B. subtilis* deposited on Al. On the other hand, it is noticed that models built from Al cannot be adapted properly to bacterial samples from STS. That is, the PLSDA model built from Al produces an accuracy of 55% and MCC of 0.10. The situation is not much improved when SVM is used. The poor modelling generalization is possibly due to limited biological replicates used in the training set of samples deposited on Al.

### 3.4. Modelling Strategy 2

#### 3.4.1. Results from Stainless Steel Substrate

Since Table 4; Table 5 both imply that the 3500–2600 cm^−1^ is most informative for discrimination between *B. subtilis* and *E. coli*, only this spectral region was considered for the following models. In this context, models were trained using one concentration level and tested on the remaining two concentrations. That is, the model was built using pixel spectra obtained from 8 replicate images of one concentration and applied on the 16 replicate images from the other two concentrations (see Table 1). Despite the same number of replicates, the number of pixels extracted from each concentration is different since higher concentration results in more detected pixels of bacteria. Specifically, 10 OD samples lead to 4020 pixels, followed by 1 OD with 1407 pixels, whereas 0.1 OD only accounts for 655 pixels (see Table 2). The modelling performance is summarized in Table 7. Obviously, the model built from one concentration works well when applied to samples of the same concentration, yet we are more interested in the results when it is applied to other concentration levels. Such outcomes are highlighted in blue-grey shading in Table 7. Using PLSDA, the model built from 10 OD produces an accuracy of 91% and MCC of 0.83 for 1 OD, yet it yields the low accuracy of 75% and MCC of 0.50 for 0.1 OD. When the PLSDA model is developed from 0.1 OD, it leads to an acceptable result for 1 OD samples with an accuracy of 89% and MCC of 0.79, and an inferior performance for 10 OD samples with an accuracy of 73% and MCC of 0.46. Meanwhile, models developed from the moderate concentration (i.e., 1 OD) demonstrate relatively good predictive capability for both 10 OD and 0.1 OD samples. That is, the accuracy and MCC for 10 OD are 87% and 0.77, respectively, and the accuracy and MCC for 0.1 OD are 82% and 0.62, respectively. In general, SVM models deliver a slightly worse modelling performance compared to PLSDA. Nevertheless, SVM modelling results imply a similar finding: the model built from 10 OD shows poor generalization when applied to 0.1 OD, and vice versa.

**Figure 6:**
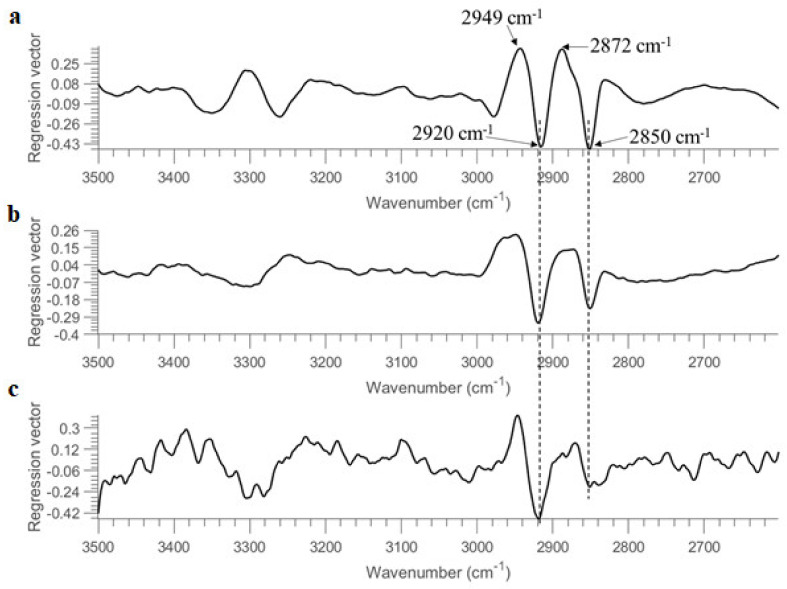
Regression vectors of PLSDA models (using 3500–2600 cm^−1^ range after SNV pre-treatment) built from (**a**) 10 OD, (**b**) 1 OD and (**c**) 0.1 OD samples deposited on STS.

The regression vectors of PLSDA models obtained from using 10 OD, 1 OD, and 0.1 OD are plotted in Figure 6. In spite of some differences, the major features of regression vectors are quite similar. The significant bands contributing to the discrimination of two bacterial strains are found at 2949 cm^−1^, 2920 cm^−1^, 2872 cm^−1,^ and 2850 cm^−1^. Bands due to ν(CH_3_) vibrations (i.e., 2949 cm^−1^ and 2872 cm^−1^) are positive, while bands of ν(CH_2_) vibrations (i.e., 2920 cm^−1^ and 2850 cm^−1^) are negative, consistent with the regression vector of PLSDA model built from the entire spectral region (see Figure 3). This opposite sign might also relate to the fact that the intensity ratio of CH_3_ groups to CH_2_ groups is higher in *B. subtilis* compared to *E.coli*, as reported in second derivative spectra (see Section 3.1).

**Figure 7:**
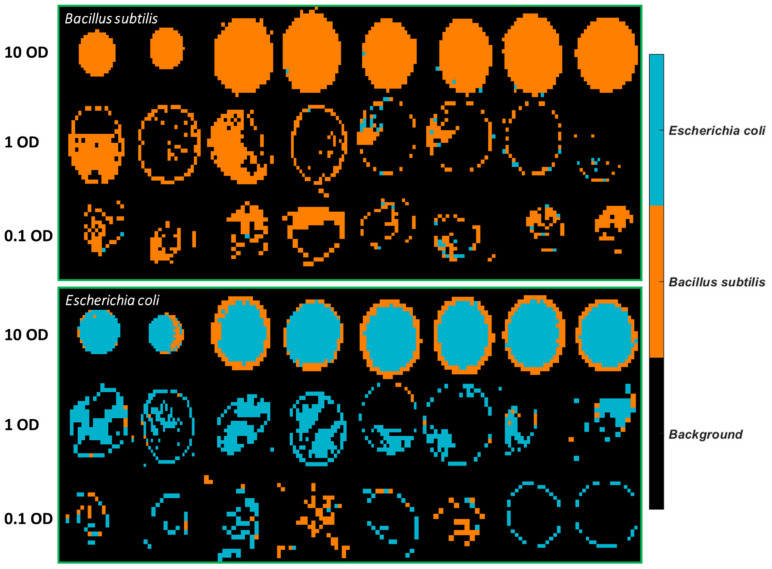
Classification maps obtained from the PLSDA model using 3500–2600 cm^−1^ for samples deposited on STS with the 1 OD samples as the training set.

The best PLSDA model using 1 OD samples as the training set was applied to develop classification maps of each sample, as shown in Figure 7. A drop of the bacterial suspension at the high concentration (10 OD) deposited on stainless steel forms a solid circular region. On the other hand, a drop of the bacterial suspension in lower concentrations either divides into smaller aggregates or tends to appear as a ring due to the so-called “coffee ring” effect. As drying proceeds, drop edges become pinned to the surface, and capillary flow outward from the centre forces suspended particles to approach the edge, leading to highly concentrated suspended particles along the edge ring [21]. Overall, more pixels of *E. coli* are wrongly classified as *B. subtilis.* The misclassified *B. subtilis* pixels are randomly distributed. In contrast, there is an abundance of misclassified pixels found at the outside layer of *E. coli* at 10 OD. The same phenomenon is not evidenced if the model was developed using half of the sample sets consisting of all concentrations as the training set (see Figure 4). Furthermore, classification maps created by using the SVM model built from 1 OD samples are also shown in Figure S10. Likewise, the misclassified pixels are distributed around the edge ring of *E. coli* at 10 OD.

**Figure 8:**
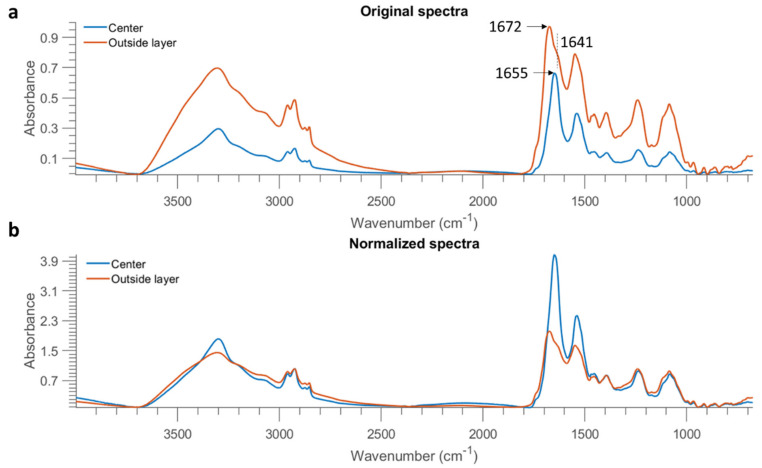
Mean spectra of the centre part and outside layer of one *E. coli* sample at 10 OD from STS (after removing baseline using asymmetric least squares smoothing) (**a**), as well as the result after normalization by dividing the intensity at 2926 cm^−1^ (**b**).

To further investigate this result, the mean spectra of the misclassified outside layer and the correctly classified centre part of *E. coli* at 10 OD were obtained and are plotted in Figure 8a. The spectrum of the outside layer demonstrates higher absorption across the entire spectral region, which is direct evidence of highly concentrated biomolecules in the edge ring. It is also observed that a shoulder at 1641 cm^−1^ emerges from the spectrum of the outside layer (as marked in the vertical dashed line of Figure 8a). The sharp absorption band at 1655 cm^−1^ originates from the amide I of α-helical structure, while the band found located at 1637 cm^−1^ is assigned to the amide I of β-sheets [9]. Therefore, this result is indicative of different protein structures due to the coffee ring effect. Normalized spectra, obtained by dividing the intensity at 2926 cm^−1^, are also obtained and exhibited in Figure 8b. A surprisingly higher proportion of amide groups are evidenced in the central part. On the contrary, the outside layer shows a higher ratio of fatty acids represented by the spectral features in the range of 3000 cm^−1^ to 2800 cm^−1^. Eales and Routh [22] studied the shape resulting after evaporation of the inkjetted droplets of a solvent containing a light-emitting polymer, and they reported that polymers with larger molecular weight mitigated the coffee ring effect because their large weight counteracted transport by the weak capillary flow. In the present study, a lesser amount of protein might transport to the edge due to its larger molecular weight compared to fatty acids, leading to more concentrated fatty acids on the outside layer but more proteins inside.

#### 3.4.2. Results from the Aluminium Substrate

Within this dataset, for each concentration level, there are four images belonging to two biological replicates acquired from samples on aluminium slides (i.e., 2 drops per biological replicate). Four images of each concentration ranging from 0.1 OD to 10 OD were used as the training set and tested on the remaining concentrations following the same modelling procedure as Section 3.4.1. It should be noted that the number of pixels identified as bacteria and used for the modelling varies for each concentration, as can be seen from Table 2. There are 2066 pixels found for 10 OD, which is remarkably reduced to 97 pixels for 1 OD and 42 pixels for 0.1 OD.

Table 8 exhibits the modelling performance acquired from using the spectral variables in 3500–2600 cm^−1^. When applying PLSDA, the use of 10 OD samples for model training results in acceptable performance for 1 OD samples but unsatisfying results for 0.1 OD samples. The PLSDA model trained by 1 OD samples produces an accuracy of 96% and MCC of 0.91 for 10 OD samples, and an accuracy of 74% and MCC of 0.46 for 0.1 OD samples. Finally, the PLSDA model developed from using 0.1 OD samples is relatively undesirable when applied to 10 OD and 1 OD samples. Regression vectors of PLSDA models trained with different concentrations are displayed in Figure S11. The correspondence of regression vectors from STS (Figure 6) and Al is not obvious. The SVM model trained by 10 OD samples works well for 1 OD samples (i.e., an accuracy of 92% and MCC of 0.85), yet it cannot be adapted properly to 0.1 OD samples (i.e., an accuracy of 62% and MCC of 0.28). Meanwhile, the SVM model trained by 1 OD samples has a strong ability to predict 10 OD samples, yet it is unable to make accurate predictions for 0.1 OD samples.

**Figure 9:**
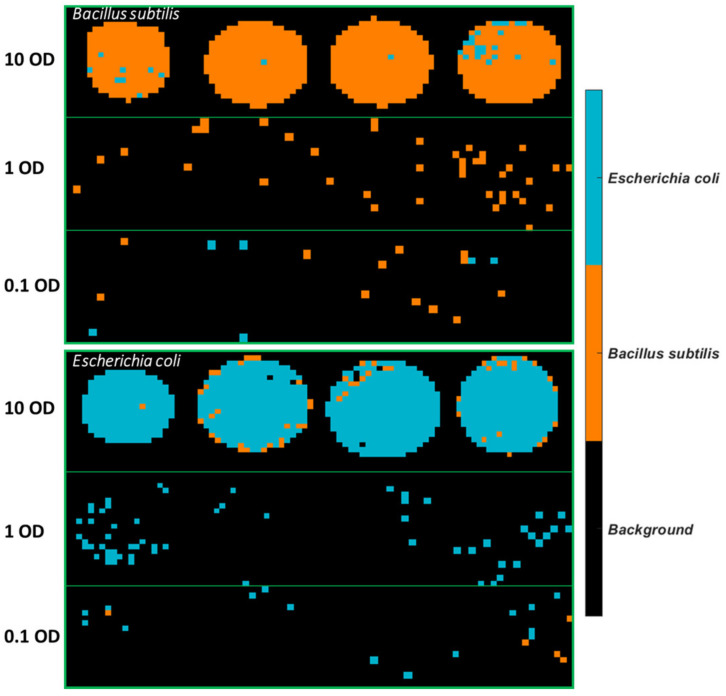
Classification maps obtained from the PLSDA model using 3500–2600 cm^−1^ for samples deposited on Al with the 1 OD samples as the training set.

**Figure 10:**
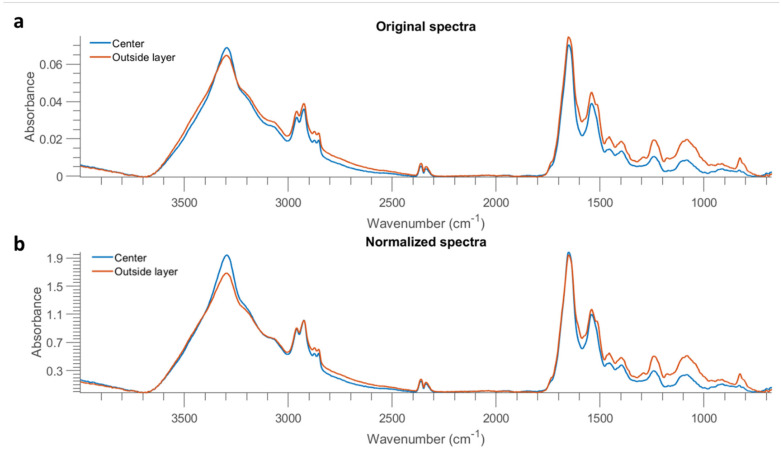
Mean spectra of the centre part and outside layer of one *E. coli* sample at 10 OD from Al (after removing baseline using asymmetric least squares smoothing) (**a**), as well as the result after normalization by dividing the intensity at 2926 cm^−1^ (**b**).

Prediction maps were generated using the best model, that is, the PLSDA model built from 1 OD samples using the 3500–2600 cm^−1^ range and are exhibited in Figure 9. Similar to the samples collected from stainless steel (Figure 7), 10 OD samples tend to appear as a solid circle. The coffee ring effect is less apparent, possibly due to the smoother surface of mirror aluminium. This concurs with research by Zhang, Chen [23] who found that the roughness of the surface strengthened the coffee ring effect because the rough structure inhibited the backflow of the capillary flow, preventing the particles’ move to the centre. Still, the majority of misclassified pixels of *E. coli* at 10 OD are found at the outside layer, which is consistent with the prediction maps of stainless steel. In the same manner, the mean spectra of the misclassified outside layer and the correctly classified centre part are plotted in Figure 10a. It is noticed that spectra obtained from Al demonstrate a smaller difference between the outside and centre part compared to that from STS, indicative of the alleviated coffee ring effect. Likewise, the outside layer reveals more concentrated biomolecules as evidenced by the stronger absorption from 3000 cm^−1^ to 675 cm^−1^. Normalized spectra (Figure 10b) suggest that a slightly higher ratio of amide groups at 1653 cm^−1^ to fatty acids are found at the central part, in accordance with Figure 8b.

## 4. Conclusions

This work investigated the potential of reflectance FTIR spectral imaging for the detection, characterisation, and discrimination between *B. subtilis* (Gram+) and *E. coli* (Gram−) cells, deposited and dried on metallic surfaces. Among the concentrations studied (0.001–10 OD), the detection limit was estimated to be 0.1 OD. Spectral profiles implicated the compositional and structural changes from different replicates due to the highly complex, dynamically changing microbial environment, cell to cell relationships and cellular activities that would lead to variations in the types and levels of cellular proteins and metabolites present. Our results suggest that it is possible to identify and discriminate two bacterial strains with a concentration as low as 0.1 OD using 10× FTIR reflectance spectral imaging. PLSDA and SVM models indicated that 3500–2600 cm^−1^ was the optimal spectral region for modelling due to no influence from atmospheric effects which devastated spectral quality at low concentrations. In addition, results proved that models built from samples with moderate (1 OD) concentration can be applied to other concentrations with good model generalization. This work could benefit the food industry through the use of a portable FTIR instrument, combined with detection and classification algorithms to enable rapid, non-destructive and real-time detection for cleaning and safety validation.

## Figures and Tables

**Table 1:** Details regarding sample replicate deposited on the stainless steel.

Concentration	Replicates	Date (Day/Month/Year)	Bacterial Strain
10 OD	8 reps	30/01/2020	EC (1rep)
		31/01/2020	EC (1rep)
		06/02/2020	BS (1rep)
		07/02/2020	BS (1rep)
		17/06/2020	EC (4reps)
		18/06/2020	BS (4reps)
		27/06/2020	EC (2reps) & BS (2reps)
1 OD	8 reps	01/07/2020	EC (4reps)
		02/07/2020	BS (4reps)
		24/09/2020	EC (2reps) & BS (2reps)
		25/09/2020	EC (2reps) & BS (2reps)
0.1 OD	8 reps	09/09/2020	EC (8 reps)
		10/09/2020	BS (8 reps)
0.001 OD	8 reps	09/09/2020	EC (8 reps)
		10/09/2020	BS (8 reps)

OD: optical density; reps: replicates; EC: *Escherichia coli*; BS: *Bacillus subtilis.*

**Table 2:** The number of pixels of each class from the two substrates that were used for modelling.

Substrate	Concentration	Number of Pixels	
		*Bacillus subtilis*	*Escherichia coli*
STS	10 OD	2037	1983
	1 OD	667	740
	0.1 OD	394	261
Al	10 OD	957	1109
	1 OD	41	56
	0.1 OD	18	24

STS: Stainless steel; Al: mirror aluminium slide.

**Table 3:** Assignment of absorbance bands for dry bacterial cells from the literature.

Wavenumber (cm^−1^)	Assignment	Possible Biomolecule Contribution	Reference
2957	ν(CH_3_) asymmetric	Fatty acids	[17]
2919	ν(CH_2_) asymmetric	Fatty acids	[17]
2872	ν(CH_3_) symmetric	Fatty acids	[9]
2852	ν(CH_2_) symmetric	Fatty acids	[9]
1741	v(C=O)	Lipid esters	[9]
~1655	Amide I	Proteins	[9]
1548	Amide II	Proteins	[9]
1457	δ(CH_2_)	Lipids, proteins	[17]
1240	ν(P=O) asymmetric	Phospholipids	[18]
~1160	δ(COP), δ(COH), ν(C–C)	DNA and RNA backbones	[9]
1200–1000	ν*(P=O) symmetric*, ν*(COC)*	Polysaccharide, DNA and RNA, phospholipids	[18]

ν: Stretch; δ: deformation

**Table 4:** Model performance to compare classifiers (PLSDA against SVM) and different spectral regions for samples deposited on stainless steel.

	Spectral Region	LVs	Training Set (3218 Pixels)				Test Set (2864 Pixels)			
			OA	MCC	Sen	Spe	OA	MCC	Sen	Spe
PLSDA	**4000–675 cm^−1^**	15	95%	0.90	0.99	0.91	90%	0.80	0.94	0.86
	1350–675 cm^−1^	8	94%	0.89	0.99	0.90	88%	0.78	0.99	0.78
	1722–910 cm^−1^	9	93%	0.87	0.95	0.92	89%	0.80	0.97	0.82
	3500–2600 cm^−1^	**10**	**99%**	**0.97**	**0.99**	**0.98**	**94%**	**0.89**	**0.94**	**0.95**
SVM	4000–675 cm^−1^	-	100%	0.99	0.99	1.00	94%	0.89	0.97	0.92
	1350–675 cm^−1^	-	99%	0.98	0.99	0.98	94%	0.88	0.97	0.90
	1722–910 cm^−1^	-	99%	0.97	0.99	0.98	94%	0.88	0.98	0.90
	3500–2600 cm^−1^	-	**99%**	**0.99**	**1.00**	**0.99**	**96%**	**0.93**	**0.96**	**0.97**

OA: overall accuracy; MCC: Matthews correlation coefficient; Sen: sensitivity; Spe: specificity.

**Table 5:** Model performance to compare classifiers (PLSDA against SVM) and different spectral regions for samples deposited on the aluminium substrate.

	Spectral Region	LVs	Training Set (1013 Pixels)				Test Set (1192 Pixels)			
			OA	MCC	Sen	Spe	OA	MCC	Sen	Spe
PLSDA	4000–675 cm^−1^	3	96%	0.95	0.98	0.97	61%	0.21	0.38	0.81
	1350–675 cm^−1^	7	99%	0.97	0.99	0.98	58%	0.16	0.56	0.60
	1722–910 cm^−1^	8	99%	0.98	1.00	0.99	51%	0.01	0.43	0.58
	3500–2600 cm^−1^	**3**	**98%**	**0.96**	**0.98**	**0.98**	**76%**	**0.53**	**0.55**	**0.93**
SVM	4000–675 cm^−1^	-	100%	1.00	1.00	1.00	69%	0.43	0.35	0.98
	1350–675 cm^−1^	-	100%	1.00	1.00	1.00	60%	0.20	0.54	0.66
	1722–910 cm^−1^	-	100%	1.00	1.00	1.00	55%	0.08	0.32	0.75
	3500–2600 cm^−1^	-	**100%**	**1.00**	**1.00**	**1.00**	**91%**	**0.82**	**0.82**	**0.98**

OA: overall accuracy; MCC: Matthews correlation coefficient; Sen: sensitivity; Spe: specificity.

**Table 6:** Modelling performance obtained from the model built from one substrate and transferred to the other substrate using 3500–2600 cm^−1^.

		Applied to	STS				Al			
	Built from		OA	MCC	Sen	Spe	OA	MCC	Sen	Spe
PLSDA	STS		-	-	-	-	71%	0.43	0.50	0.89
	Al		55%	0.10	0.40	0.70	-	-	-	-
SVM	STS		-	-	-	-	82%	0.66	0.61	0.99
	Al		57%	0.15	0.50	0.65	-	-	-	-

OA: overall accuracy; MCC: Matthews correlation coefficient; Sen: sensitivity; Spe: specificity.

**Table 7:** Modelling performance of PLSDA and SVM classifiers built from one concentration and applied to other concentrations (deposited on STS) using 3500–2600 cm^−1^.

		Applied to		10 OD				1 OD				0.1 OD			
	Built from		LVs	OA	MCC	Sen	Spe	OA	MCC	Sen	Spe	OA	MCC	Sen	Spe
**PLSDA**	10 OD		10	100%	1.00	1.00	1.00	91%	0.83	0.98	0.84	75%	0.50	0.98	0.40
	1 OD		6	87%	0.77	1.00	0.75	95%	0.91	0.95	0.95	82%	0.62	0.94	0.62
	0.1 OD		7	73%	0.46	0.72	0.73	89%	0.79	0.85	0.93	93%	0.85	0.95	0.90
**SVM**	10 OD		-	100%	1.00	1.00	1.00	89%	0.81	0.99	0.81	75%	0.52	1.00	0.38
	1 OD		-	86%	0.75	0.99	0.73	98%	0.96	0.98	0.98	83%	0.65	0.95	0.66
	0.1 OD		-	69%	0.40	0.51	0.87	87%	0.75	0.79	0.94	95%	0.90	0.98	0.92

OA: overall accuracy; MCC: Matthews correlation coefficient; Sen: sensitivity; Spe: specificity.

**Table 8:** Modelling performance of PLSDA and SVM classifiers built from one concentration and applied to other concentrations (deposited on Al) using 3500–2600 cm^−1^.

		Applied to		10 OD				1 OD				0.1 OD			
	Built from		LVs	OA	MCC	Sen	Spe	OA	MCC	Sen	Spe	OA	MCC	Sen	Spe
**PLSDA**	10 OD		4	99%	0.98	0.99	0.98	87%	0.72	0.83	0.89	55%	0.16	0.78	0.38
	1 OD		9	96%	0.91	0.97	0.95	100%	1.00	1.00	1.00	74%	0.46	0.67	0.79
	0.1 OD		5	57%	0.14	0.11	0.96	72%	0.42	0.56	0.84	100%	1.00	1.00	1.00
**SVM**	10 OD		-	100%	1.00	1.00	1.00	92%	0.85	1.00	0.86	62%	0.28	0.78	0.50
	1 OD		-	96%	0.92	0.95	0.96	100%	1.00	1.00	1.00	50%	0.08	0.78	0.29
	0.1 OD		-	57%	0.17	0.09	0.99	55%	0.06	0.44	0.63	100%	1.00	1.00	1.00

OA: overall accuracy; MCC: Matthews correlation coefficient; Sen: sensitivity; Spe: specificity.

## Data Availability

The data presented in this study are available on request from the corresponding author.

## Supplementary Materials

The following are available online. Table S1. Cell counts obtained for selected dry cell samples deposited on stainless steel and aluminium. Figure S1. Microscopic images of EC and BS at 10 OD, 1 OD and 0.1 OD. Figure S1. A picture of an empty mirror aluminium slide. The wells are labelled with numbers. For a certain bacterial strain, 10 OD concentrations are located at 1 and 7, 1 OD at 2 and 8, 0.1 OD at 3 and 9, 0.01 OD at 4 and 10, 0.001 OD at 5 and 11, while 6 and 12 are empty. Figure S3. Mean spectra of each replicate sample at 10 OD deposited on stainless steel. Rep: replicate. Figure S4. Pixel spectra (including both bacterial and stainless steel pixels) without any pre-processing extracted from one sample image of each concentration deposited on stainless steel. Figure S5. Pixel spectra of stainless steel. Figure S6. Mean spectra of all bacterial cells from concentrations of 10 OD, 1 OD and 0.1 OD deposited on stainless steel. Figure S7. Normalized mean spectra of all bacterial cells from concentrations of 10 OD, 1 OD and 0.1 OD deposited on stainless steel. Normalization is performed by dividing the intensity at 2926 cm−1. Figure S8. Pixel spectra of mirror aluminium slide. Figure S9. Pixel spectra without any pre-processing extracted from one sample of each concentration deposited on mirror aluminium slide. Figure S10. Classification maps obtained from the SVM model using 3500–2600 cm−1 for samples deposited on STS with the Set 2 (1 OD) data as the training set. Figure S11. Regression vectors of PLSDA models (using 3500–2600 cm−1 range after SNV pre-treatment) built from (A) 10 OD, (B) 1 OD and (C) 0.1 OD samples deposited on Al.
