# Supplementary material for: Characterisation and Classification of Foodborne Bacteria Using Reflectance FTIR Microscopic Imaging
Source: Molecules. 2021 Oct 19;26(20):6318. doi: 10.3390/molecules26206318 (PMC8541507; doi:10.3390/molecules26206318)
Supplement: Supplementary file 1 [file molecules-26-06318-s001.zip › molecules-1395308-supplementary.pdf]

molecules

Supplementary Material

Characterisation and Classification of Foodborne Bacteria Using Reflectance FTIR Microscopic Imaging

Jun-Li Xu [1], Ana Herrero-Langreo [1], Sakshi Lamba [2,3], Mariateresa Ferone [2], Amalia G. M. Scannell [2,3], Vicky Caponigro [1,3] and Aoife A. Gowen [1,*]

1 School of Biosystems and Food Engineering, University College Dublin, Belfield, Dublin 4, Ireland; junli.xu@ucd.ie (J.-L.X.); ana.herrero-langreo@ucd.ie (A.H.-L.); vicky.caponigro@ucd.ie (V.C.)
2 School of Agriculture and Food Science, University College Dublin, Belfield,Dublin 4, Ireland; sakshi.lamba@ucdconnect.ie (S.L.); mariateresa.ferone@ucd.ie (M.F.); amalia.scannell@ucd.ie (A.G.M.S.)
3 Institute of Food and Health, University College Dublin, Belfield, Dublin 4, Ireland
* Correspondence: aoife.gowen@ucd.ie.

Table S1. Cell counts obtained for selected dry cell samples deposited on stainless steel and aluminium.

Sample set	OD	Cell counts (logCFU/ml)	
		EC	BS
On mirror stainless steel (09–10 Sep 2020)	0.1	5.75	3.72
	0.001	4.45	1.00
On mirror aluminium slide (23–24 Sep 2020)	10	8.60–8.70	7.00–7.60
	1	7.91–7.85	6.48–6.66
	0.1	6.81–6.86	3.34–3.68
	0.01	5.41–5.58	1.78–2.04
	0.001	3.52–3.60	1.00–1.30

Note: OD: optical density; EC: Escherichia coli; BS: Bacillus subtilis.

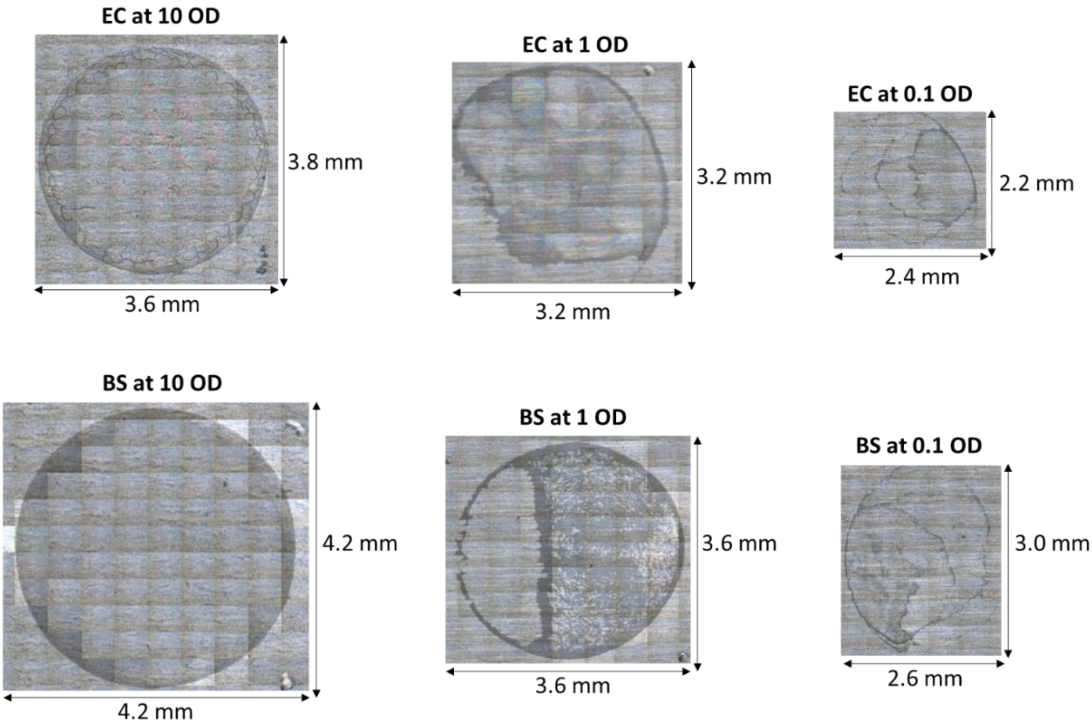

Figure S1. Microscopic images of EC and BS at 10 OD, 1 OD and 0.1 OD.

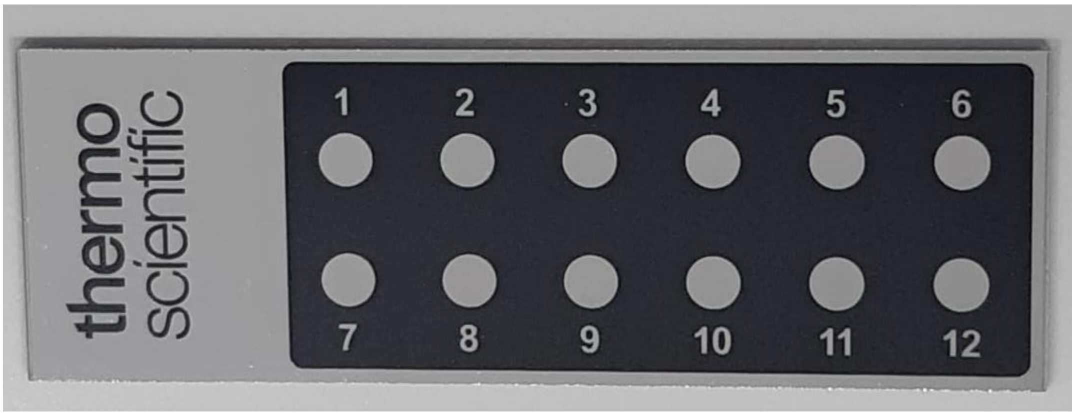

Figure S2. A picture of an empty mirror aluminium slide. The wells are labelled with numbers. For a certain bacterial strain, 10 OD concentration are located at 1 and 7, 1 OD at 2 and 8, 0.1 OD at 3 and 9, 0.01 OD at 4 and 10, 0.001 OD at 5 and 11, while 6 and 12 are empty.

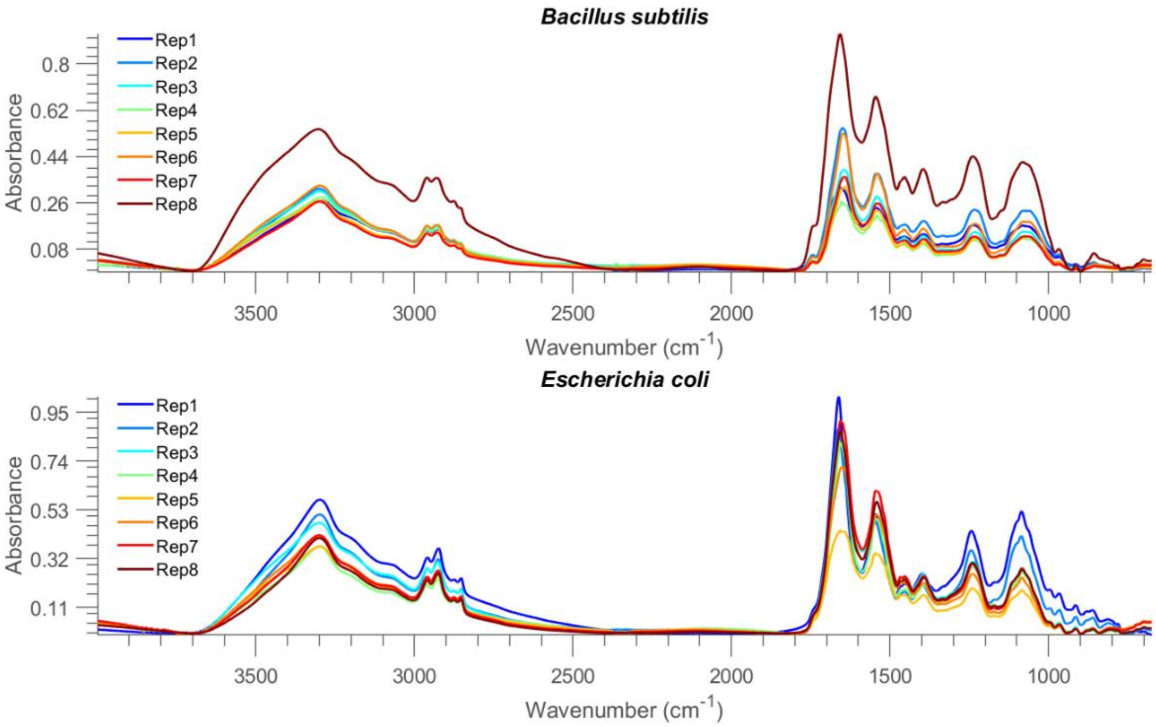

Figure S3. Mean spectra of each replicate sample at 10 OD deposited on stainless steel. Rep: replicate.

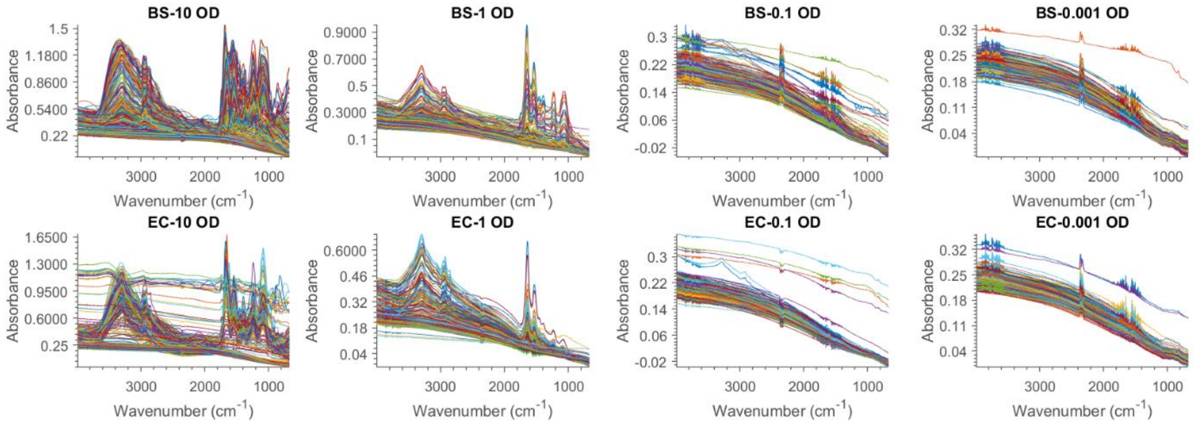

Figure S4. Pixel spectra (including both bacterial and stainless steel pixels) without any pre-processing extracted from one sample image of each concentration deposited on stainless steel.

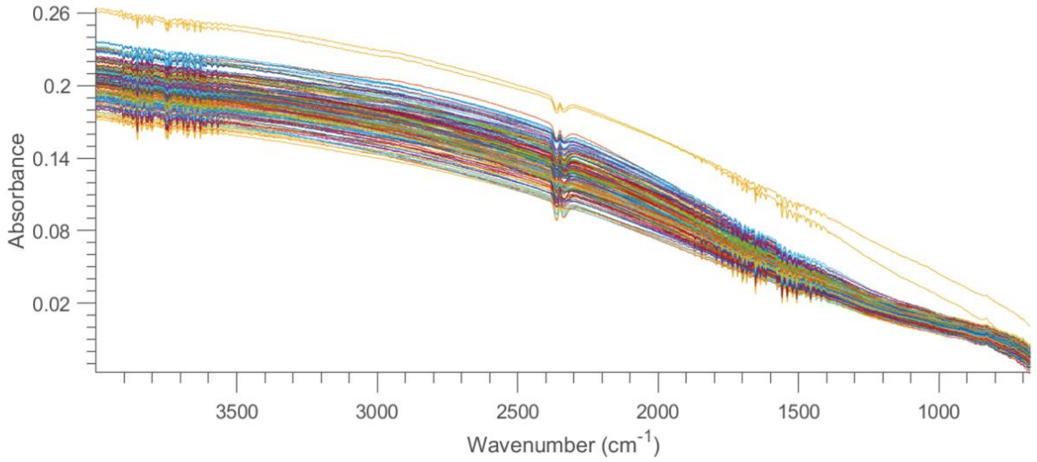

Figure S5. Pixel spectra of stainless steel.

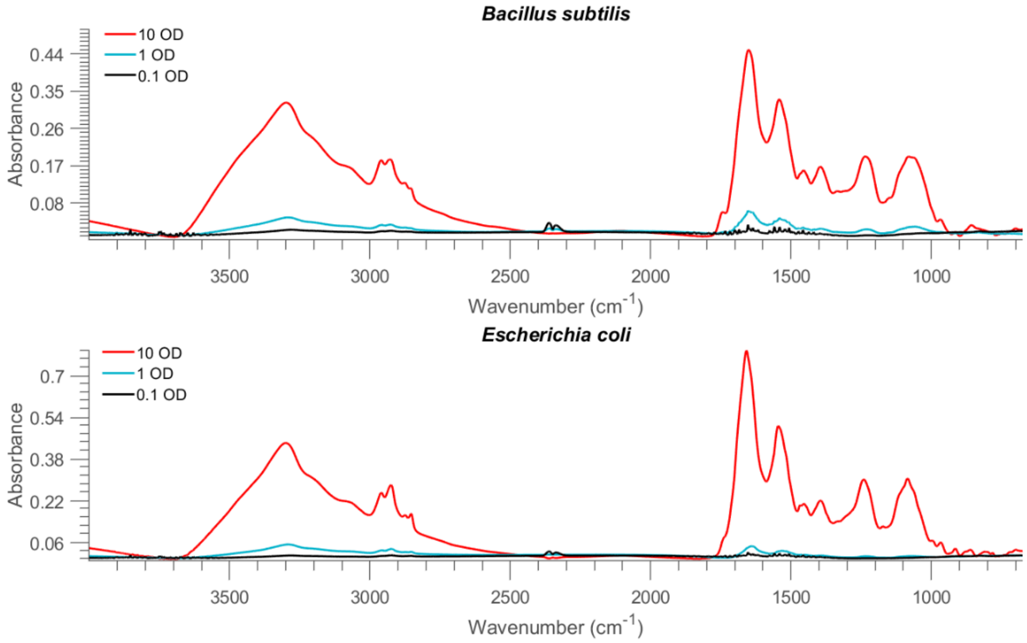

Figure S6. Mean spectra of all bacterial cells from concentrations of 10 OD, 1 OD and 0.1 OD deposited on stainless steel.

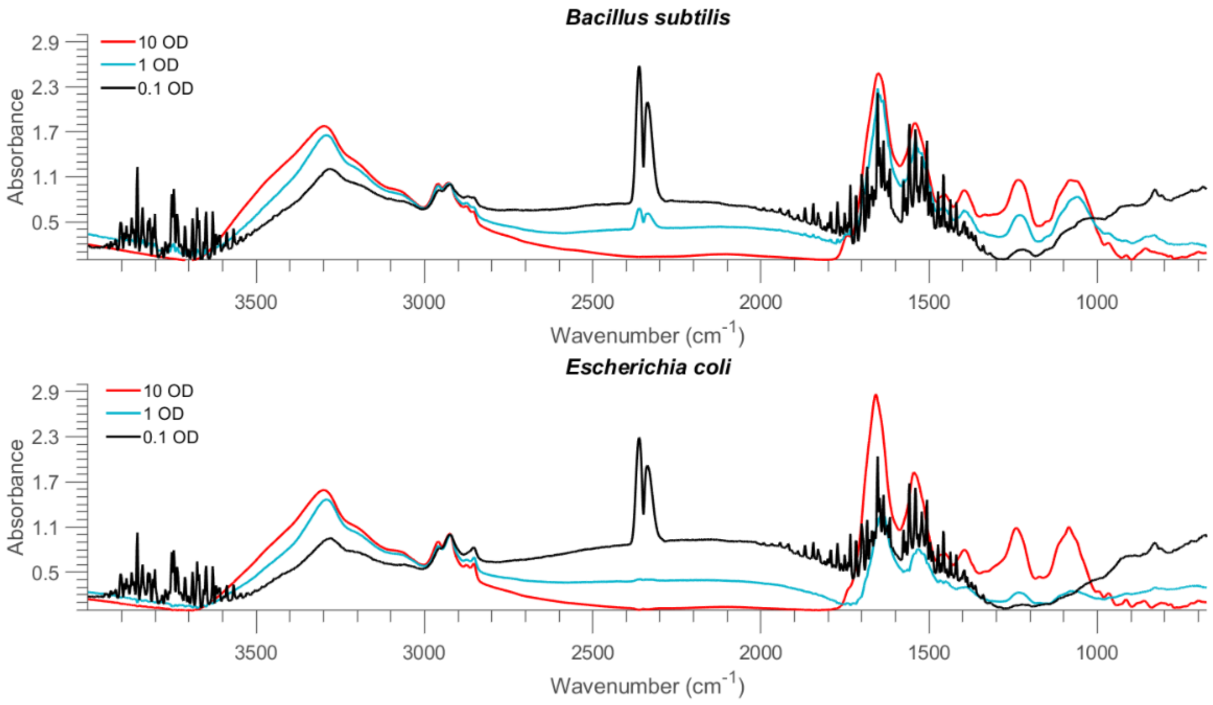

Figure S7. Normalized mean spectra of all bacterial cells from concentrations of 10 OD, 1 OD and 0.1 OD deposited on stainless steel. Normalization is performed by dividing the intensity at 2926 cm^{-1}.

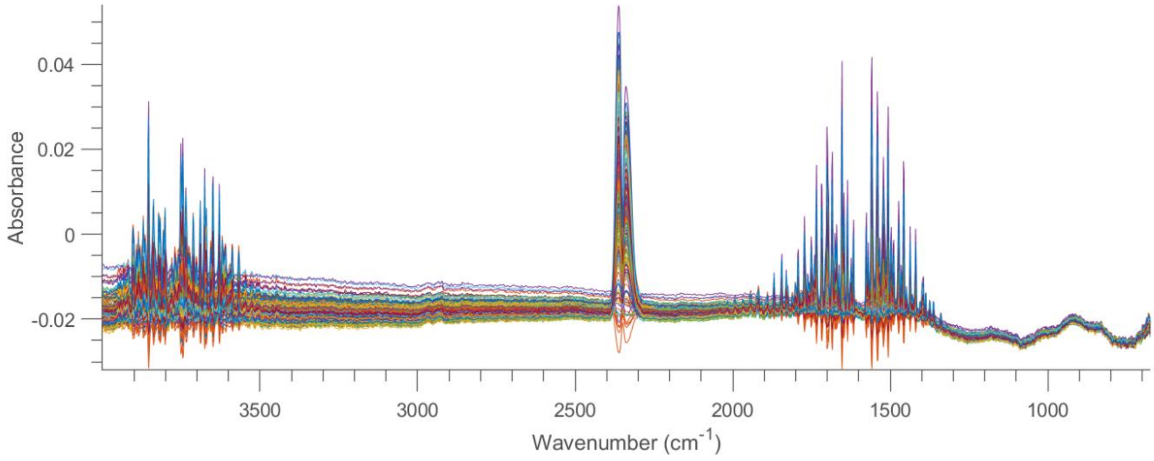

Figure S8. Pixel spectra of mirror aluminium slide.

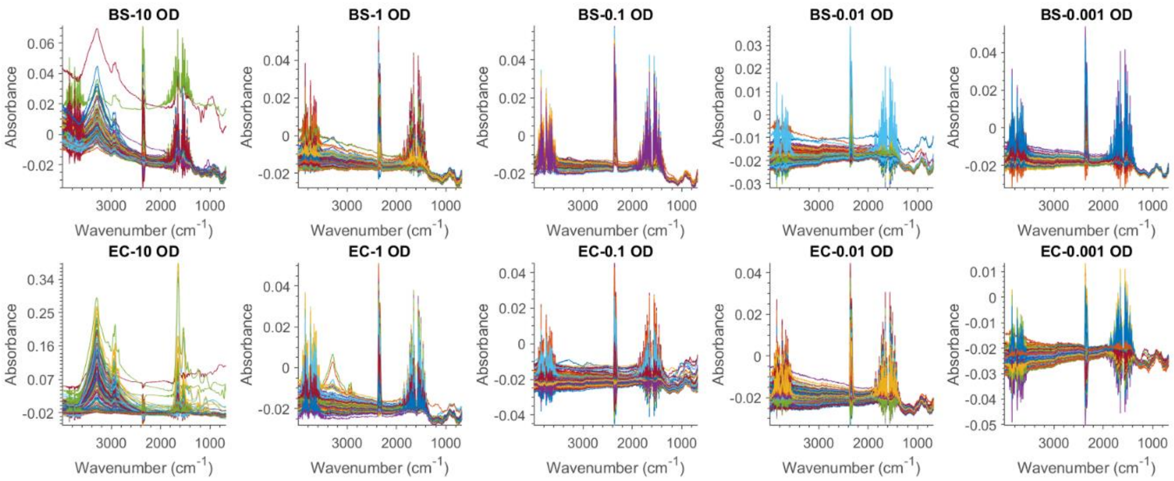

Figure S9. Pixel spectra without any pre-processing extracted from one sample of each concentration deposited on mirror aluminium slide.

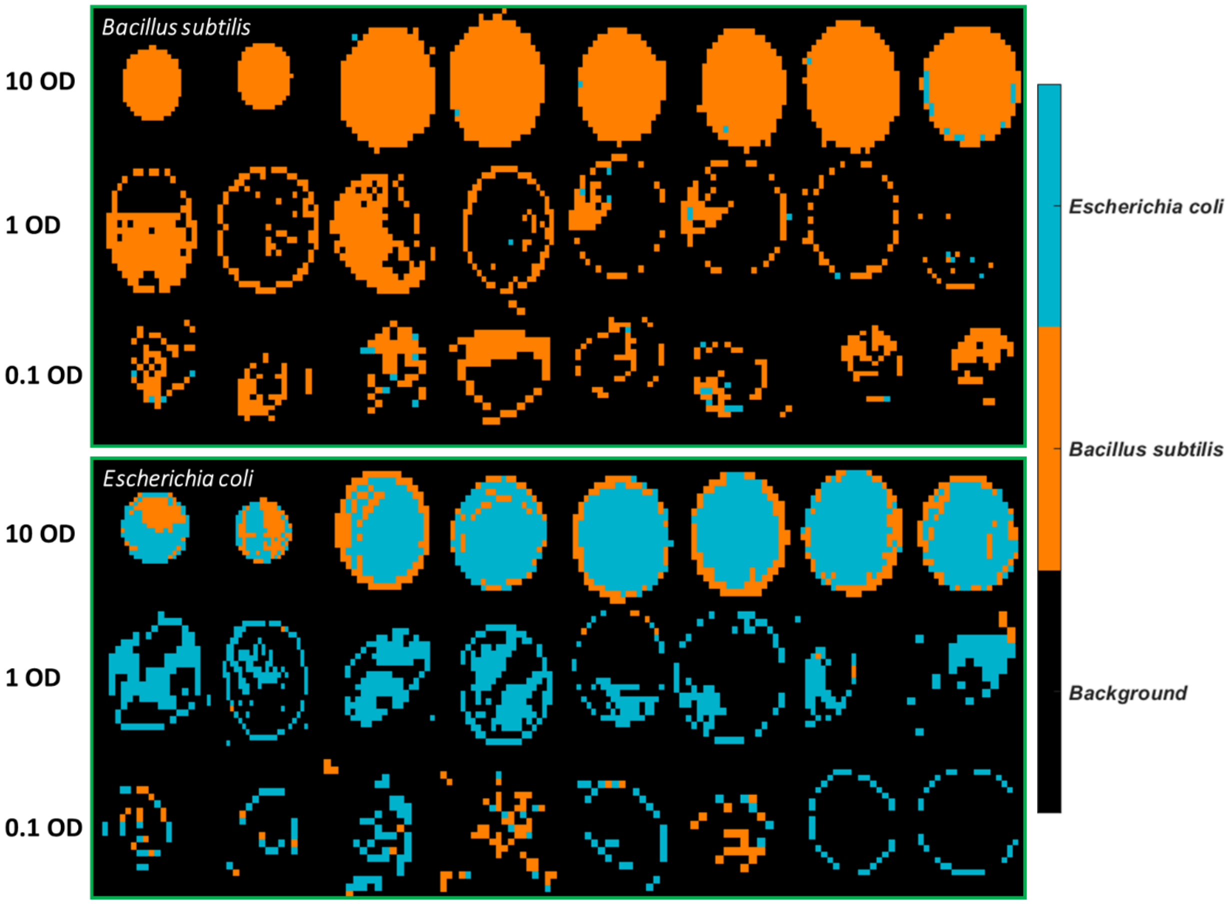

Figure S10. Classification maps obtained from the SVM model using 3500–2600 cm^{-1} for samples deposited on STS with the Set 2 (1 OD) data as the training set. BS: *Bacillus subtilis*; EC: *Escherichia coli*.

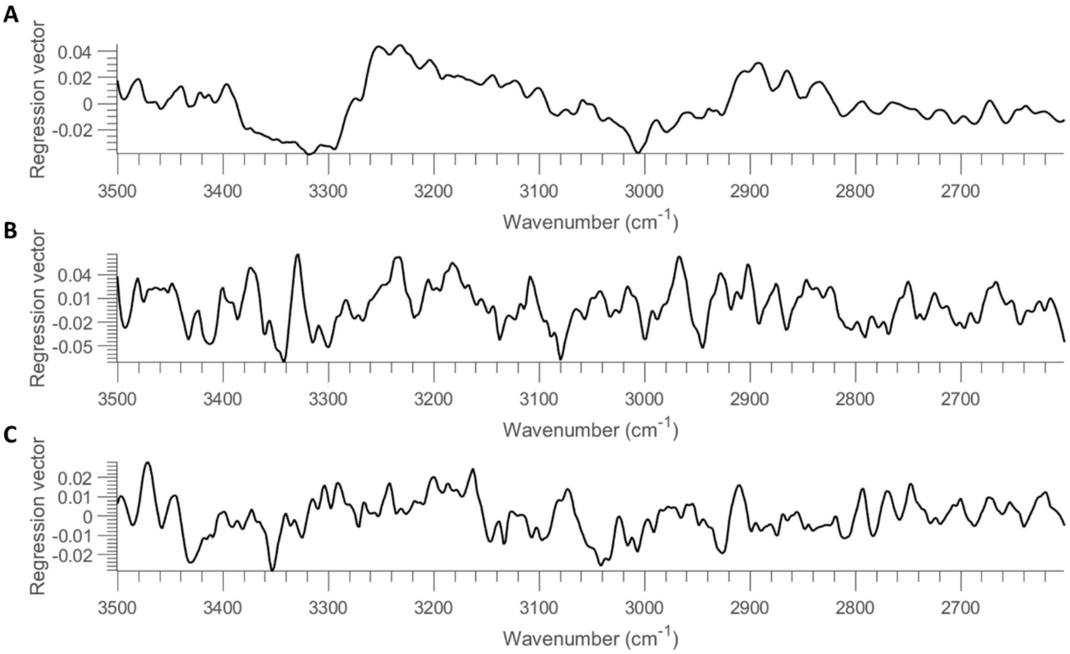

Figure S11. Regression vectors of PLSDA models (using 3500–2600 cm^{-1} range after SNV pre-treatment) built from (A) 10 OD, (B) 1 OD and (C) 0.1 OD samples deposited on Al.
